# The importance of planning CT-based imaging features for machine learning-based prediction of pain response

**DOI:** 10.1038/s41598-023-43768-6

**Published:** 2023-10-13

**Authors:** Óscar Llorián-Salvador, Joachim Akhgar, Steffi Pigorsch, Kai Borm, Stefan Münch, Denise Bernhardt, Burkhard Rost, Miguel A. Andrade-Navarro, Stephanie E. Combs, Jan C. Peeken

**Affiliations:** 1 Department of Radiation Oncology, Klinikum Rechts der Isar, Technical University of Munich (TUM), Ismaninger Straße 22, 81675 Munich, Germany; 2 Department for Bioinformatics and Computational Biology, Informatik 12, Technical University of Munich (TUM), Boltzmannstraße 3, 85748 Garching, Germany; 3 Institute of Organismic and Molecular Evolution, Johannes Gutenberg University Mainz, Hanns-Dieter-Hüsch-Weg 15, 55128 Mainz, Germany; 4 Department of Radiation Sciences (DRS), Institute of Radiation Medicine (IRM), Helmholtz Zentrum, 85764 München, Germany; 5 Deutsches Konsortium für Translationale Krebsforschung (DKTK), Partner Site Munich, 69120 Heidelberg, Germany

**Keywords:** Bone metastases, Machine learning

## Abstract

Patients suffering from painful spinal bone metastases (PSBMs) often undergo palliative radiation therapy (RT), with an efficacy of approximately two thirds of patients. In this exploratory investigation, we assessed the effectiveness of machine learning (ML) models trained on radiomics, semantic and clinical features to estimate complete pain response. Gross tumour volumes (GTV) and clinical target volumes (CTV) of 261 PSBMs were segmented on planning computed tomography (CT) scans. Radiomics, semantic and clinical features were collected for all patients. Random forest (RFC) and support vector machine (SVM) classifiers were compared using repeated nested cross-validation. The best radiomics classifier was trained on CTV with an area under the receiver-operator curve (AUROC) of 0.62 ± 0.01 (RFC; 95% confidence interval). The semantic model achieved a comparable AUROC of 0.63 ± 0.01 (RFC), significantly below the clinical model (SVM, AUROC: 0.80 ± 0.01); and slightly lower than the spinal instability neoplastic score (SINS; LR, AUROC: 0.65 ± 0.01). A combined model did not improve performance (AUROC: 0,74 ± 0,01). We could demonstrate that radiomics and semantic analyses of planning CTs allowed for limited prediction of therapy response to palliative RT. ML predictions based on established clinical parameters achieved the best results.

## Introduction

Bone metastasis, a common complication in oncology, poses significant difficulties in predicting pain response for patients. Machine learning (ML) techniques have been often used to address different oncological challenges, given the innovative approach they offer^1–5^.

There is a significant amount of cancer research based on ML techniques, applying different ML algorithms such as support vector machines (SVMs) and random forest classifiers (RFCs)^6–8^. One field that has experienced a rapid growth over the last few years thanks to the use of ML techniques to extract information from these features is radiomics^9–13^.

Radiomics data can be used for training ML models to predict clinical or biological outcomes^14–16^. Radiomics has been employed across different cancer to anticipate survival, disease prognosis, tumour response, molecular abnormalities, as well as identifying metastases or regions of invasive tumour growth^17–28^.

Nonetheless, the use of radiomics feature analysis to predict non-tumour radiotherapy (RT) response hasn’t been extensively explored. A few investigations have examined the projection of RT-related complications, including xerostomia, pneumonitis or proctitis^29–31^. In the context of bone metastasis, unfortunately, there remains a dearth of studies, with only a few focusing on the prediction of non-tumour RT responses^32–35^. However, there are general limitations for ML-related studies in this domain, where dataset sizes are significantly smaller than expected for the more common ML algorithms. Without the proper statistical strengthening of the resampling technique, this problem can potentially lead to wider error margins and, on occasions, overoptimistic results. Nevertheless, these studies underscore the importance of further research in this domain.

Painful spinal bone metastases (PSBMs) are regularly treated by palliative RT. About two thirds of the patients experience a partial or complete response in terms of pain reduction^34^. The role of biomarkers and personalised RT in PSBM cases has become increasingly prominent^36–38^. Clinical parameters, such as age, Karnofsky performance score (KPS), use of opioids or cancer histology (e.g. breast or prostate cancer), show limited predictive capabilities to identify patients that profit from palliative RT^33^. The Spinal instability neoplastic score (SINS) has been developed by the Spine Oncology Study Group to assess instability of spinal bone metastases^39^. At the same time, the SINS provides a semantic tool to predict pain response to RT^34^.

In this retrospective study we sought to determine the potential of ML-based prediction of RT therapy response of PSBM. Besides clinical features, we investigated whether CT-based radiomics features and semantic features can be used to predict pain response, as well. The best strategy for the definition of volumes of interest (VOI) in regard to macroscopic or microscopic metastatic expansion was assessed for radiomics feature extraction. In order to statistically strengthen and produce more robust results, SVM, RFC and logistic regression (LR) models were trained, evaluated and compared using repeated nested cross-validation, stratifying the splits for multiple patient samples.

## Materials and methods

### Clinical data curation

Patient records of all (n = 491) patients treated with palliative RT for bone metastases between 2009 and 2017 at our institution were analysed. Patients with non-spinal metastases, previous interventions (e.g., surgical stabilization or kyphoplasty) or RT, haematological bone manifestations, and missing information regarding pain response were excluded (Figure S1 for a patient workflow).

Patient demographics were assessed for each patient (Table 1 for characteristics of patients, RT and metastatic disease). Clinical parameters previously shown to be associated with pain response such as KPS, age, use of opioids, and histology (breast cancer, non-small cell lung cancer (NSCLC) and others) were determined and used as input for the clinical ML models (Table S1 for the exact distribution of histologies)^33,34,40,41^. These clinical features were measured prior to RT. Histology, as the only categorical value present in the clinical data, was encoded into three dummy binary features.

**Table 1:** List of semantic features.

Feature	Possible values
Imaging—Bone reaction	Blastic reaction
	Mixed reaction (lytic/blastic)
	Lytic reaction
Soft tissue component	Yes
	No
GTV classification	Any portion of vertebral body
	Lateralized within body
	Diffuse within body
	Body + unilateral pedicle
	Body + bilateral pedicle/transverse processBody
	Unilateral pedicle
	Unilateral lamina
	Spinous process
Posterolateral involvement of the spinal elements	Bilateral
	Unilateral
	None of the above
Vertebral body collapse	> 50% collapse
	< 50% collapse
	No collapse with > 50% body involved
	None of the above
Location	Junctional
	Mobile
	Semirigid
	Rigid

Pain response was rated retrospectively on the basis of patient records following the “international consensus on palliative radiotherapy endpoints for future clinical trials in bone metastases” at the first follow-up visit 6 weeks after RT^42^: complete response: “pain score of 0 at treated site with no concomitant increase in analgesic intake”, partial response: “﻿Pain reduction of at least 2 at the treated site (scale of 0 to 10) without analgesic increase, or analgesic reduction of at least 25% without pain increase”, pain progression: “﻿increase in pain score of at least 2 or increase of analgesics of at least 25%”, and indeterminate response or “no response”: “no response or any response not captured by the other categories”. In both complete and partial responses, patient-rated worst pain measures were used.

Planning CT images acquisition parameter and orientation were performed via axial reconstruction of cross-sectional images using a Siemens Somatom Emotion 16 with 3 mm slice thickness and 0.98 mm × 0.98 mm resolution (Table S2 shows all CT image acquisition parameters). The SINS was determined by visual assessment of planning CTs following the definition of the Spine Oncology Study Group^39^. Visual assessment was performed by JA and supervised by JCP. The SINS was used for ML modelling both as a discrete variable and as a binary variable using a threshold of 7. Approval from the institutional review board of the Technical University of Munich hospital was received (reference number 466/16 s). All patients were treated after informed consent. All experiments were performed in accordance with local legal regulation allowing retrospective data analysis.

### Definition of VOIs

For each metastasis, two separate VOI definitions were segmented on the planning CT scans using Eclipse 13.0 (Varian Medical Systems, Palo Alto, USA) (Table S2 for acquisition parameters). First, the visible blastic and/or lytic gross tumour volume (GTV) including any adjacent soft-tissue component was manually segmented. Secondly, a clinical target volume (CTV) considering potential microscopic spread was segmented following the International Spine Radiosurgery Consortium Consensus Guidelines for Target Volume Definition in Spinal Stereotactic Radiosurgery^43^. The segmentation process of the CTV was performed manually by HA and supervised by JCP.

### Radiomics feature extraction

**Figure 1:**
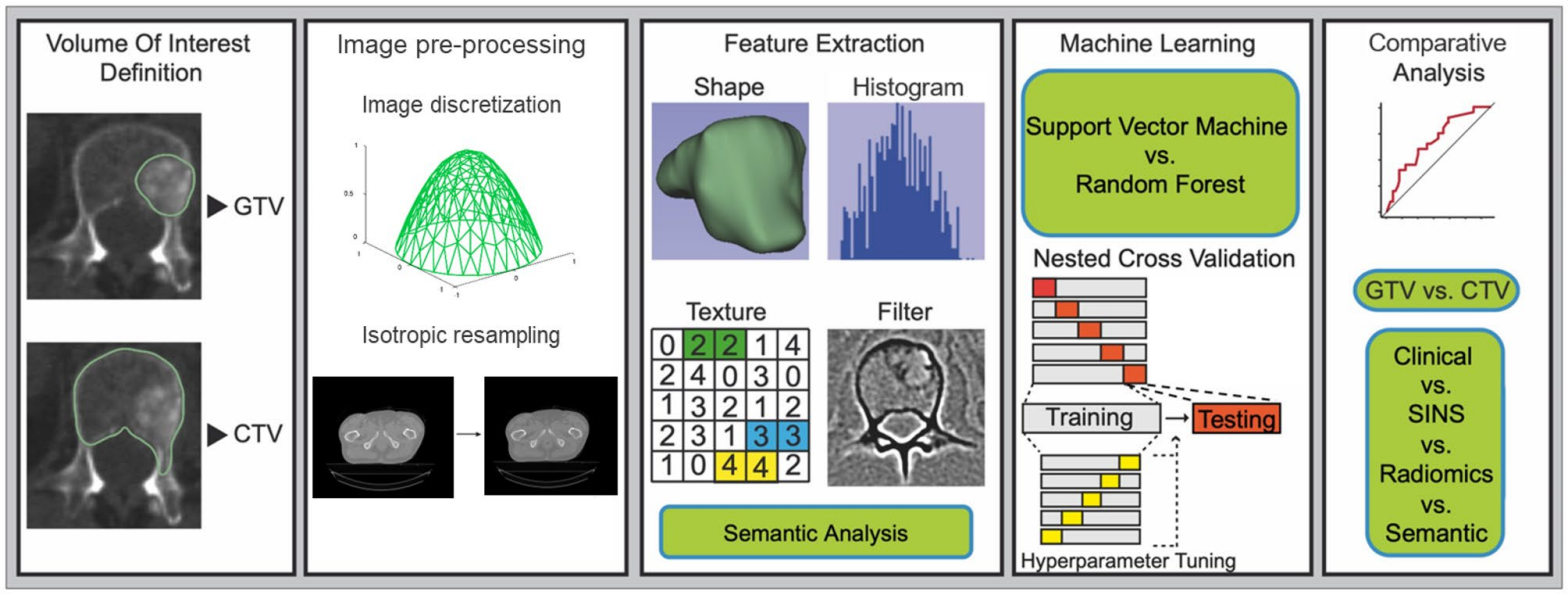
Workflow.

Pre-processing and radiomics feature extraction were performed using the pyRadiomics library (version 2.0) in Python (version 3.6.4) (Fig. 1 for study workflow)^44^. For pre-processing, a fixed bin width of 20 was used for image discretization. The intensity ranges between all patients were 218–2083 HU (Min–Max). In accordance to earlier studies, and the pyRadiomics guidelines for images with similar characteristics, a bin width of 20 was chosen in order to retain a bin number in the range of 30–130^45^. 105 radiomics features, including shape, first-order, and texture features were computed from the original image. Texture features were calculated in 3D. Gray Level Co-occurrence Matrix (GLCM) and Gray Level Run Length Matrix (GLRLM) texture features were calculated separately for each direction and then averaged. All extracted features were computed according to the “image biomarker standardization initiative” guidelines (Table S3)^46^.

### Semantic features extraction

Semantic features from the SINS score and other imaging descriptors were determined by an MD student (JA) and controlled by a radiation oncology resident with 3 years of experience (JCP) (see Table 1 for a complete listing). The resident trained the medical student on a per-patient basis for the first 20 patients together. Subsequently random patients were controlled and all patients with more difficult allocation to a semantic group.

Many of the semantic features are part of the SINS score (Location, Bone Reaction, Vertebral Collapse 50%, posterolateral involvement) which has been correlated with pain response^34^. For GTV classification, the extent of metastasis is part of the CTV definition recommendations and has not yet been associated with response. Soft Tissue Component was once tested in one study without showing an association with tumour response^47^.

### Machine learning modelling

The number of patients was filtered by removing incomplete entries, taking the intersection of patients with all CTV, GTV, semantic, clinical and SINS data, and performing outlier detection. This resulted in a dataset with 230 pre-processed PSBM with known outcome. For feature reduction, both redundancy reduction and feature correlation to the prediction target were taken into consideration with the Maximum Relevance – Minimum Redundancy (MRMR) algorithm (mrmr-selection library, version 0.2.2)^48^. For all feature sets larger than 15 throughout this study, the best 15 features were selected to be used by the respective ML algorithms, so that the number of features for every model amount to up to 10% of the number of samples.

Given the small dataset size and to ensure a correct hyperparameter optimization, nested fivefold cross-validation was applied to train and validate the ML models. However, multiple samples coming from the same patient, present in the same data subsample, may lead to biased and over-optimistic results. To offset this, cross-validation splits were stratified by patient ID: this way, there is an even distribution of such samples across the 5 splits in either fold. In order to correct the moderate class imbalance (negative to positive ratio of 3.11:1), Synthetic Minority Oversampling Technique (SMOTE) was used (imbalanced-learn library, version 0.8.0). To avoid overfitting by class repetition, random minority class oversampling was complemented with random majority class undersampling. The normalisation, feature selection and class imbalance correction steps were performed in the inner fold of the nested cross-validation to avoid data leakage and bias. Nested cross-validation was repeated for 50 iterations, for a total of 250 aggregated models, to increase the statistical strength of the results.

Hyperparameter optimization was performed via exhaustive grid search in the inner fold of the nested cross-validation, using balanced accuracy (BA) as the optimization criteria. SVM and RFC were used for training on the multivariable datasets i.e., both radiomic segmentations, the semantic and the clinical feature sets; and Logistic Regression (LR) was used for the analysis of SINS. For SVM, the hyperparameters optimized were C, gamma (when applicable), the degree (when applicable) and the kernel used. For RFC, the hyperparameters optimized were max_features, max_depth, min_samples_split, min_samples_leaf, bootstrap, and criterion. The only hyperparameter optimized for LR was C. A summary of the optimized hyperparameters of the best radiomics, combined and overall modelling strategies can be found in Table S12. All models come from the scikit-learn library^49^ (version 0.24.2). Firstly, these models were trained on both segmentation modes: CTV and GTV to assess their predictive quality against a binary prediction target (Table 3): complete pain response (complete response vs partial response/indeterminate response/no response/pain progression). Results were compared to determine the best modelling strategy. The best model was then compared to clinical, SINS, and semantic models (Table 4). Finally, multiple combined models were devised to assess whether combined models performed better (Tables 5 and S6).

The importance given by models to their features was recorded in order to analyse the feature importance for all models developed. Since it is not possible to track the weight of features for non-linear kernels in SVM, only the percentage of feature selection was shown. For RFC models, this importance is shown as the Gini Importance or mean decrease in impurity of the nodes (the higher, the more important).

### Statistical analysis

Given the small dataset size and, therefore, unclear class distribution, Min–max normalisation was performed to scale all features (scikit-learn library, version 0.24.2), while retaining the same distribution. Outlier detection is performed before the nested cross-validation (where normalization, feature selection and class balancing are conducted) to avoid extreme values from affecting the distribution of the data (scikit-learn library, version 0.24.2). All error margins are reported as standard errors with a coefficient of 1.96 for a confidence interval covering 95% of the observations. All models were evaluated, principally, using the Area Under the Receiver-Operator Curve (AUROC). In addition, BA, F1 score and Matthews Correlation Coefficient (MCC) were secondarily examined. The most important AUROC comparisons have been quantitatively evaluated with the Mann–Whitney U test to determine whether they follow the same distribution (null hypothesis), using a p-value of 0.05 for a 95% confidence interval. Given the dataset size limitation, the models trained on either radiomics segmentation did not use the intersection of all available patients but all available. This has prevented the possibility of using a DeLong test for quantitative AUROC evaluation. Statistical analysis and radiomics model building were performed using Python (version 3.7) and conducted by OL-S.

## Results

### Pain response to RT

A retrospective cohort of 90 patients with a total of 267 PSBM fitted the inclusion and exclusion criteria in our institution (Figure S1 for a patient workflow). Mammary carcinoma, prostate carcinoma and NSCLC were the three most frequent (63%) cancer types (Table 2 for patient characteristics and Table S1 for a distribution of all cancer histologies). There was a median of two PSBMs per patient with a total of 41 solitary PSBMs. Partial and complete pain response retrospectively assessed from patient files was achieved in 33% and 52% of patients, respectively.

**Table 2:** Characteristics of patients, radiotherapy and metastatic disease with complete information.

Patient characteristic	Complete response (n = 30 p)	Partial or no response (n = 60 p)	*p* value^a^
Gender: Male	14 p (43%)	29 p (48%)	0.82
Gender: Female	16 p (57%)	31 p (52%)	
Age	m 66 (r 26–88)	m 66 (r 30–87)	0.74
Karnofsky Performance score	m 70 (r 60–100)	m 80 (r 30–90)	0.34
Opioid medication	16 p (57%)	37 p (62%)	0.50
Tumour type	Mammary/prostate carcinoma: 11 p (37%)	Mammary/prostate carcinoma: 31 p (52%)	0.30
	NSCLC: 7 p (23%)	NSCLC: 8 p (13%)	
	Others: 12 p (40%)	Others: 21 p (35%)	
Partial response	–	47 p (78%)	–
Overall survival	m 5.5 months	m 7.5 months	0.90
	(r 0.7–55.8 months)	(r 0.1–68.1 months)	
Radiotherapy			
Single dose	m 3 (r 2–8)	m 3 (r 2–8)	0.54
Total dose	m 33 (r 8–44)	m 30 (r 8–45)	0.10
Number of fractions	m 10 (r 1–22)	m 10 (r 1–19)	0.45
Bone metastases			
Number of metastases	65	196	
Number of metastases per patient	m 1.5 (r 1–6)	m 2.5 (r 1–10)	0.055
Previous RT	0 p (0%)	0 p (0%)	–
Localization	Sacrum: 8 p (12%)	Sacrum: 17 p (9%)	0.26
	Lumbar: 34 p (52%)	Lumbar: 83 p (42%)	
	Thoracic: 21 p (32%)	Thoracic: 83 p (42%)	
	Cervical: 2 p (3%)	Cervical: 13 p (7%)	
Bone reaction	Blastic: 15 p (23%)	Blastic: 56 p (29%)	0.03
	Lytic: 31p (48%)	Lytic: 28 p (14%)	
	Mixed: 19 p (29%)	Mixed: 112 p (57%)	
Soft tissue component	25 p (38%)	48 p (25%)	0.08
Extent of metastasis^b^	vertebral body: 17 p (26%)	vertebral body: 54 p (28%)	0.03
	body/pedicle: 4 p (6%)	body/pedicle: 9 p (5%)	
	`body/pedicle/transverse process: 2 p (3%)	body/pedicle/transverse process: 8 p (4%)	
	Unilateral pedicle: 23 p (35%)	Unilateral pedicle: 35 p (18%)	
	Unilateral lamina: 18 p (28%)	Unilateral lamina: 88 p (45%)	
	Spinous process: 1 p (2%)	Spinous process: 3 p (2%)	
SINS	m 7 (3–14)	m 8 (0–15)	0.02

m: median, p: patients, r: range, SINS: Spinal Instability Neoplastic Score.

^a^Wilcoxon rank sum test for continuous and ordinal variables, Fisher’s exact test for nominal variables, log rank test for comparison of survival times. The significance level for these tests has been Bonferroni corrected for family-wise error rate, resulting in an adjusted significance level of 3.33e-3 for an original alpha of 0.05.

^b^Following the Gross Tumour Volume (GTV) classification of the ﻿International Spine Radiosurgery Consortium^1^.

### Determination of the best VOI for radiomics analysis and modelling strategy

**Figure 2:**
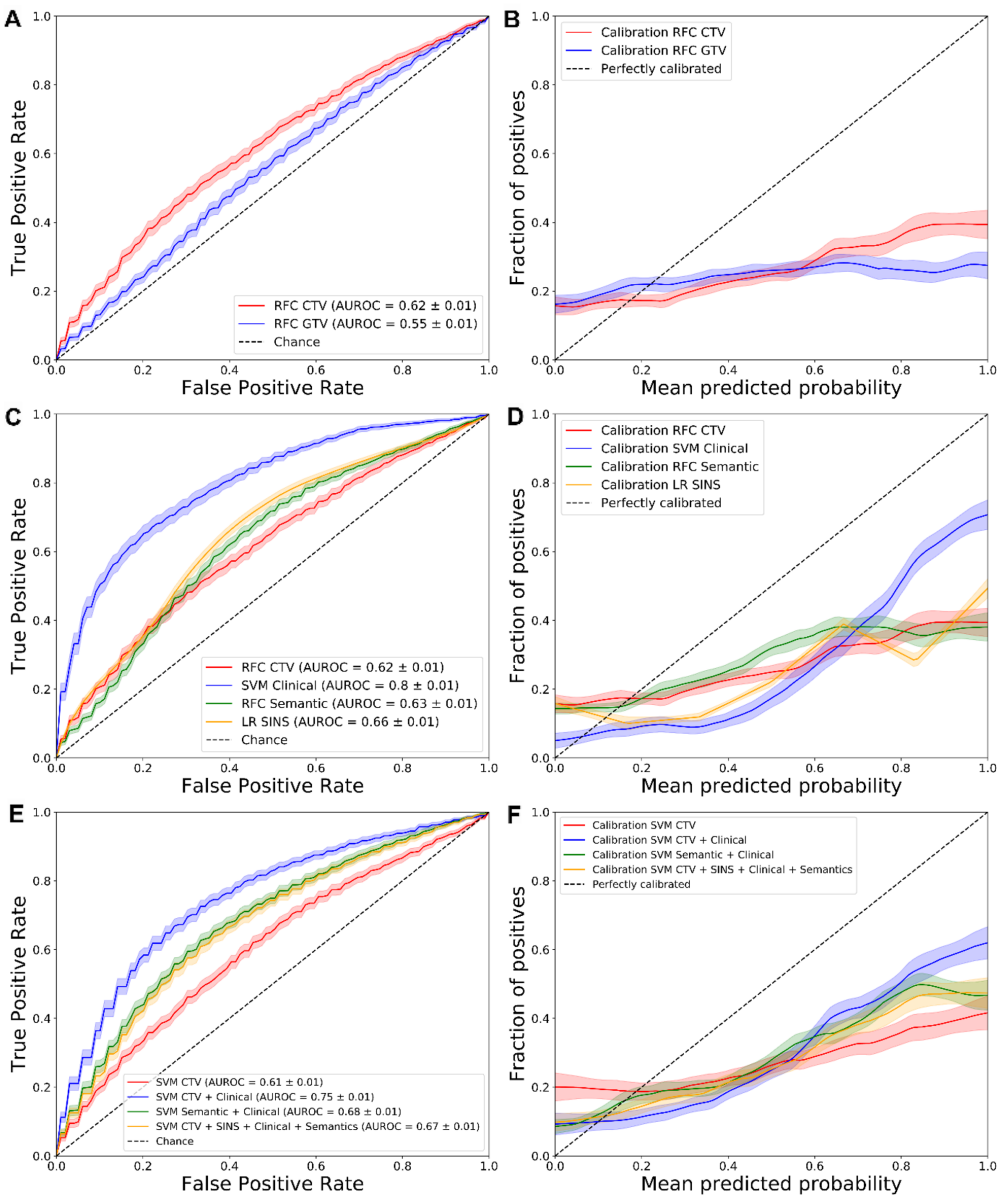
Receiver operator characteristic (ROC) and Calibration curves for the comparisons of different segmentation modes (**A**, **B**), clinical baseline, semantic and SINS features (**C**, **D**), and combined models (**E**, **F**).

The best performing model was a RFC trained on the CTV radiomics segmentation, with the highest overall scores (AUROC: 0.62 ± 0.01) (Table 3 for outcome metrics and Fig. 2 for ROC and calibration curves). While the data was imbalanced towards the negative class (no complete pain response), it has performed better when predicting the true positive class (complete pain response), as it can be seen in the confusion matrix provided (Figure S3). This is further confirmed by a higher specificity (0.72) than sensitivity (0.44). While the RFC reached the highest performance, the SVM results were more stable. The best segmentation mode was CTV, with higher performance regardless of the modelling strategy. Lastly, the Mann–Whitney U test comparing the AUROC distributions of the best performing models from Table 3 had a *p* value of 4.70-e13, therefore confirming that the AUROC results are statistically different.

**Table 3:** AUROC, BA, F1 Score and MCC for the best modelling algorithms trained on both radiomics segmentation modes (GTV and CTV).

Segmentation	Model	AUROC	BA	F1	MCC
GTV	SVM	0.58 ± 0.01	0.54 ± 0.02	0.33 ± 0.03	0.08 ± 0.04
CTV	RFC	0.62 ± 0.01	0.58 ± 0.02	0.37 ± 0.03	0.15 ± 0.04

### Comparison to clinical baseline, semantic and SINS models

The best segmentation mode among the radiomics models (CTV) was then compared to the clinical, the semantic and the SINS models (Table 4 and Fig. 2).

**Table 4:** AUROC, BA, F1 Score and MCC for the best models, comparing the best radiomics model to the semantic features, clinical baseline and SINS variable.

Data	Model	AUROC	BA	F1	MCC
CTV	RFC	0.62 ± 0.01	0.58 ± 0.02	0.37 ± 0.03	0.15 ± 0.04
Semantic	RFC	0.63 ± 0.01	0.58 ± 0.02	0.39 ± 0.03	0.16 ± 0.04
Clinical	SVM	0.80 ± 0.01	0.72 ± 0.03	0.56 ± 0.05	0.43 ± 0.06
SINS	LR	0.65 ± 0.01	0.58 ± 0.03	0.36 ± 0.05	0.16 ± 0.06
SINS (binary)		0.54 ± 0.01	0.52 ± 0.03	0.19 ± 0.05	0.04 ± 0.06

The semantic features, on the other hand, achieved almost identical results to the best radiomics segmentation: none performed statistically better. Lastly, a LR trained only on the SINS variable achieved very different results: SINS (binarized) performed very close to random, with a poor classification quality (MCC: 0.04 ± 0.06); on the other hand, the non-binarized SINS model performed similar to the CTV-based radiomics segmentation model but higher AUROC (0.65 ± 0.01).

The clinical ML model outperformed all other models regardless of the modelling algorithm with statistical significance (Table S5 and Figure S2). The best clinical model (SVM) predicted pain response with a BA of 0.72 ± 0.03 and an AUROC of 0.80 ± 0.01. Similar to the best performing radiomics model, while the data was moderately imbalanced towards the negative class (no complete pain response), the best performing model overall has shown a better prediction quality when evaluating the true positive class (complete pain response), as it can be seen in the according confusion matrix provided (Figure S4). This is further confirmed by a higher specificity (0.82) than sensitivity (0.63). The AUROC distribution of the SVM model trained on clinical data has been compared, with a Mann–Whitney U test, to that of the other models shown in Table 4 (except to the LR model trained on SINS (binary)). The p-values were 4.21e-57, 3.02e-59 and 2.15e-49 respectively, confirming that the AUROC values of the clinical model are statistically and significantly better.

Given the limited features that the SINS and clinical datasets comprised, their respective prediction models showed a wider standard error on scores where greater variance was expected (F1 and MCC).

### Benefits by combining imaging and clinical features

The SVM was evaluated on the possible performance increase by combining the best radiomics model (CTV), clinical, SINS, and semantic features (Table 4, Fig. 2 and Figure S2).

The best performance with combined models was achieved with a SVM trained on CTV and clinical data (AUROC: 0.75 ± 0.01). The addition of non-binarized SINS did not significantly affect the performance of any combined model. An SVM model trained on all data (CTV, non-binarized SINS, clinical and semantic features) outperformed one using only radiomics data; however, it was significantly worse than the best combined model. The Mann–Whitney U test comparing the AUROC distribution of the best semantic model (RFC), and a combined model of semantic and clinical data (SVM), resulted in a p-value of 1.12e-05, therefore confirming that the combined clinical models perform significantly better than semantic features alone.

Interestingly, a model trained only on semantic and clinical features achieved the same performance level as the combined model with all available features (AUROC: 0.68 ± 0.01 and 0.67 ± 0.01, respectively). None of the combined features outperformed the SVM using clinical features.

### Feature importance

Feature importance was estimated for SVM and RFC trained on CTV, clinical baseline, semantic, and combined sets of data (Tables S8 to S11). None of the features from the CTV models were selected in all of the 250 cases. On the other hand, the top 15 features for both CTV models (SVM and RFC) were highly homogeneous, sharing the same top three texture features. The most important semantic features were the extent of the GTV along with features also used in the SINS score (e.g., lytic bone lesions and bilateral posterolateral involvement of the spinal element).

The mean decrease in impurity of the RFC nodes, overall, showed low values, with most being below 0.1. However, clinical features achieved a significantly higher feature importance, which is in concordance with the higher performance of those models.

The combined SVM model of CTV, Clinical, SINS and Semantic features showed the same low importance values, with almost no feature selected in 100% of the cases. The feature that was selected most often, while also retaining high importance, was the clinical feature “Tumour Type: Breast Cancer” followed by predominantly semantic and clinical features. Although the majority of all features in the combined model were radiomics (105 of 135), only four of the 12 most predictive features were radiomic, while most of them were semantic.

## Discussion

In this exploratory analysis we analysed the potential of ML models to predict pain response to RT of PSBM. CT-based radiomics machine learning models predicted pain response better than random. CTV-based outperformed GTV-based models; semantic and SINS-based models outperformed random, and clinical models performed best, with SVM at the peak. The combination of radiomics features with clinical data significantly increased performance compared to the radiomics baseline. This combination, however, did not match models using only clinical features. The addition of the SINS feature neither affected the radiomics nor the combined model. The feature importance of all radiomics features showed low levels of mean impurity decrease in RFC. Texture features have proven to be the most important predictors, achieving both high percentages of feature selection and high importance scorings. Only clinical features have shown a high importance level, while they were also often consistently selected.

In our modelling approach, we compared two established ML models. Both models achieved competitive results. The best ML model, for radiomics data, was the RFC by a small but statistically significant margin (Tables 3 and S4). However, the SVMs performed better in some situations, mainly for other metrics such as the BA and F1 score. In addition, the SVM models achieved the best results when trained on clinical data, and performed better than the RFCs for the combined data models (Table 5 and S6). The SVMs achieved more consistent results when trained on radiomics features: these models had more competent performances than the RFCs when trained on features with, in principle, less useful information. Given the low importance of these features, these results indicate that the SVM is more resilient to selected features with poor importance. This is further confirmed when analysing the combined models: combined SVM models achieved consistently better performances than RFCs.

**Table 5:** AUROC, BA, F1 Score and MCC for SVM models trained on the combination of radiomic, clinical, SINS and semantic features.

Data	Model	AUROC	BA	F1	MCC
CTV + SINS	SVM	0.61 ± 0.01	0.57 ± 0.02	0.36 ± 0.04	0.13 ± 0.04
CTV + Clinical		0.75 ± 0.01	0.69 ± 0.02	0.52 ± 0.03	0.35 ± 0.04
Semantic + SINS		0.62 ± 0.01	0.58 ± 0.02	0.39 ± 0.03	0.15 ± 0.04
Semantic + Clinical		0.68 ± 0.01	0.63 ± 0.02	0.45 ± 0.03	0.24 ± 0.04
CTV + SINS + Clinical + Semantic		0.67 ± 0.01	0.62 ± 0.02	0.44 ± 0.03	0.22 ± 0.04

We have compared the predictive performance of multiple sets of data: two radiomics segmentation modes, clinical, semantic and SINS features. The only model that did not achieve better than random results was LR trained only on SINS (binarized; Table 4). This is to be expected: by binarizing the SINS variable, important information, that can be learnt by either model, is lost. Combined models that used clinical data had an expected performance increase compared to their respective baselines (Table 5 and S6). However, these combined models performed worse than a clinical only model: this indicates that the addition of features that are not important to the model can have a negative impact on its performance, by making it difficult for the model to identify patterns in the data. This is further confirmed by the decrease in feature importance of the clinical features when comparing them alone and in a combined model (Tables S9 and S11, respectively).

All radiomics features have shown low feature importance, which can be explained by a possible low correlation to the prediction target. This is also consistent with the fact that none were selected in any of the 250 cases. In addition, only 10 of all 105 features were selected by MRMR at least 50% of the time. This high variance when selecting features is potentially due to their low correlation towards the *complete pain response* outcome variable. On the other hand, clinical features have shown more than thrice higher feature importance towards the outcome variable, and were selected in nearly all cases when used in combined modelling (Tables S9 and S11).

Multiple previous publications have analysed factors related to pain response following RT of bone metastasis. An early retrospective study by Arcangeli et al. demonstrated that pain response depended on patients’ performance status and specific histology. NSCLC patients were shown to have a worse response to RT than patients with other cancer origins^40^. This was reproduced by Nyguen et al. demonstrating a favourable response for patients with prostate and mammary carcinoma^41^. Location and pain level before therapy appeared not to influence radiation response^32,50^. These results were validated in a large prospective trial with 956 patients by Westhoff et al. Next to the aforementioned clinical factors, the use of opioids and absence of visceral metastases were positively predictive for RT response^33^. However, the multivariate model achieved only limited predictive capacity with a C-statistic of 0.56.

Van Velden et al. conducted a further prospective trial comparing the predictive performance of the SINS with clinical parameters^34^. SINS appeared to be significantly associated with complete response after adjustment for gender, tumour type and performance status. Adding SINS to the clinical parameters increased the AUROC for the prediction of complete response from 0.68 to 0.78. In our study, SINS as training data proved to perform better than random (Table 4). However, adding SINS to other datasets did not increase their performance significantly (Table 5 and S6). Combining clinical and SINS data, the overall performance was significantly better than the radiomics models (SVM and RFC AUROCS: 0.73 ± 0.01 and 0.75 ± 0.01, respectively), albeit inferior to the clinical models (Table S7). The performance difference of a combined model of clinical and SINS features between Van Velden et al. study and this exploratory analysis can be attributed to a number of reasons. Firstly, in this study, pain response was assessed retrospectively, which can potentially explain the different performance of the models towards the outcome variable. Secondly, the different proportion of metastases localization, and the presence of cervical cases, may affect the SINS, given the higher instability that some locations may entail. Thirdly, the current study employed SVMs and RFCs as models trained on clinical and SINS features, which are distinct from the multivariate logistic regression used in the previous study. Fourthly, in Van Velden et al. study it is not directly explained what resampling technique the authors have used. A difference in the resampling technique can potentially impact the prediction performances due to larger training sizes, therefore leading to over-optimistic results in some cases. Lastly, the clinical features used in both studies are significantly different, leading to different model performances (AUROC values of 0.68 and 0.80 in the previous study and ours, respectively). Therefore, the room for improvement for the SINS variable in a combined model with clinical features be substantially different.

In our study, we compared two potential modes of segmentation. Although the predictive performance was overall similar, the CTV-based segmentations were superior for both ML models. In contrast to the GTV, the CTV segmentation included vertebra compartments that are at risk of microscopic infiltration^43^. This additional information may have improved the predictive power. Texture features were the most important radiomics features. Such features may capture texture and intensity heterogeneity that may be associated with cell density within the bone marrow. Analysis of magnetic resonance imaging data may be more suitable to quantify such changes. Recently, one other publication has analysed the potential of radiomics-based prediction of pain response^35^. The authors trained a random forest model on a single centre cohort of 69 patients using leave-one-out cross-validation. While their clinical model showed an inferior performance with an AUC of 0.70, the radiomics model was able to predict pain response with a superior AUC of 0.82. There are several reasons that may explain these differences in performance. First, the authors applied only the simplistic double-layer split into train and test set (through their leave-one-out cross-validation), instead of the more adequate triple-layer split into train, validation and test set. Consequently, the authors optimized their model on the same patients used for assessing performance, thereby opening the door to data leakage. Such leakage often leads to substantial over-estimates of performance. In contrast, our nested cross-validation results included repeated testing independent of hyperparameter optimization guaranteeing more unbiased results. Second, the authors used a different set of VOIs. Instead of a GTV or specific CTV, the authors used the spinal canal, the complete vertebra and the vertebra plus a one-centimetre margin as VOIs. So far, it remains unclear what segmentation strategy may be optimal. Third, the authors trained their model for “any pain response” instead of “complete response” which may also explain a difference in performance by having a broader prediction target. Taken together with our results, both studies could demonstrate prediction of pain response better than random albeit with different predictive power against the background of a significantly different study design.

Besides quantitative radiomics image analysis, semantic features extraction constitutes an alternative “manual” way to extract information from medical images^51^. For prediction of pain response of PSBM Mitera et al. evaluated semantic imaging features in 33 patients^47^. The authors did not find any association of semantic imaging features to pain response. Semantic features included pathological fractures, kyphosis and anatomic extent of tumour. However, the study was limited by the use of a large number of semantic features and a relatively small number of patients. For instance, the known predictive factor age did not correlate with response either. Our study has shown that, with a larger training set, it is possible to achieve better than random prediction results when training either ML algorithm with semantic data, with RFC performing best (AUROC: 0.63 ± 0.01; Table 4). It is important to note that the SINS score in itself is a score combining multiple semantic features. We used these features complemented with other additional variables. The SINS score, however, performed better than the semantic model, demonstrating that the important features are already included in the SINS score.

There are several limitations to our study. First, pain response was assessed retrospectively. Due to non-standardized or incomplete reporting of pain response determination, it may have been error prone. To allow a standardised assessment we followed the recommendation of the International Spine Radiosurgery Consortium Consensus Guidelines^43^. Patients with “indeterminate response” were excluded from analysis. This may have conferred a selection bias as missing information may be associated with confounding factors such as low KPS or early death. Secondly, in patients with multiple PBMS each metastasis was treated as a separate sample. The outcome, however, was equal between all metastases of a specific patient. Information on which specific metastases contributed to symptomatic pain remained elusive. To prevent data leakage and bias, stratified cross-validation was performed, guaranteeing that multiple samples from the same patient were evenly distributed across all splits. Thirdly, our study was of monocentric nature with a lack of an external validation set. To compensate for this, we applied nested cross-validation and repeated the process 50 times to increase the statistical strength of the results. We believe that our exploratory analysis allows the assessment of the general possibility of RT response prediction and a comparison to established factors.

## Conclusions

To conclude, in this exploratory work we were able to demonstrate a predictive value of established clinical factors using machine learning for the prediction of complete pain response to palliative radiotherapy in patients with painful spinal bone metastases. CT-based radiomics and semantic machine learning models performed better than random but sub-optimally. The SINS score performed slightly better than both, and models trained on a combination of the available datasets performed even better. Using exclusively clinical features as input, however, outperformed all other models. Upon inspection of the radiomics and clinical features, their importance and selection frequency confirmed the higher predictive quality of the latter, with a more than three-fold decrease in mean impurity. Thus, CT-based radiomics features did not present supplementary value beyond models trained solely on clinical features.

### Supplementary Information

Supplementary Information.

## Data Availability

All data and code used in this research is available upon contact of the correspondence author (Jan C. Peeken, jan.peeken@tum.de) and in concordance to the ethics committee.
