# Supplementary material for: The importance of planning CT-based imaging features for machine learning-based prediction of pain response
Source: Sci Rep. 2023 Oct 13;13:17427. doi: 10.1038/s41598-023-43768-6 (PMC10576053; doi:10.1038/s41598-023-43768-6)
Supplement: Supplementary file 1 — Supplementary Information. [file 41598_2023_43768_MOESM1_ESM.pdf]

Supplemental Material

SUPPLEMENTAL TABLES 2
TABLE S1 2
TABLE S2 3
TABLE S3 4
TABLE S4 10
TABLE S5 10
TABLE S6 10
TABLE S7 11
TABLE S8 11
TABLE S9 11
TABLE S10 12
TABLE S11 13
TABLE S12 13
SUPPLEMENTAL FIGURES 15
FIGURE S1 15
FIGURE S2 15
FIGURE S3 17
FIGURE S4 17
REFERENCES 18

Supplemental Tables

TABLE S1
Histology distribution

Relative proportion of all histology types ranked by their frequency.

Histology	Proportion of patients
Mammary carcinoma	25 (28 %)
Prostate carcinoma	17 (19 %)
Non-Small Cell Lung Cancer	15 (16 %)
Urothelium cancer	6 (7 %)
Pancreatic carcinoma	3 (3 %)
Renal cell carcinoma	9 (10 %)
Cancer of unknown primary	1 (1 %)
Rectal carcinoma	1 (1 %)
Cholangiocellular carcinoma	1 (1 %)
Parotic carcinoma	1 (1 %)
Esophageal carcinoma	2 (2 %)
Thymoma	1 (1 %)
Hepatocellular carcinoma	1 (1 %)
Gliosarcoma	1 (1 %)
Small Cell Lung Cancer	1 (1 %)
Gastric cancer	2 (2 %)
Thyroid cancer	1 (1 %)
Adrenal gland carcinoma	1 (1 %)
Hypopharyngeal carcinoma	1 (1 %)

TABLE S2
CT acquisition Parameters

Parameter	
Scanner type	Somatom Emotion 16 Siemens (Erlangen, Germany)
Axial scan dimensions	512 x 512 pixel
Pixel Spacing	0.98 mm x 0.98 mm
Slice thickness	3 mm
Kernel	B31s
Voltage	130 KVP
X ray tube Current	398.5 (94-650)

Abbreviations: CT: computed tomography

TABLE S3
Extracted radiomics features

All extracted features were computed according to the "image biomarker standardization initiative" (IBSI) guidelines [1]. The pyRadiomics package (version 2.0) implemented in python (version 3.6.4) was used for feature extraction [2]. For pre-processing, a fixed bin width of 20 was used for image discretization. Isotropic resampling was performed to a voxel size of 1x1x1 mm using Bspline interpolation.

	**Shape Features**
1.)	Volume
2.)	Surface Area
3.)	Surface Volume Area
4.)	Sphericity
5.)	Spherical Disproportion
6.)	Maximum 3D Diameter
7.)	Maximum 2D Diameter Slice
8.)	Maximum 2D Diameter Column
9.)	Maximum 2D Diameter Row
10.)	Major Axis
11.)	Minor Axis
12.)	Least Axis
13.)	Elongation
14.)	Flatness
	**First Order Features**
1.)	Energy
2.)	Intensity Histogram Entropy
3.)	Minimum
4.)	10th Percentile
5.)	90th Percentile
6.)	Maximum
7.)	Mean
8.)	Median
9.)	Interquartile Range

10.)	Range
11.)	Mean Absolute Deviation (MAD)
12.)	Robust Mean Absolute Deviation (rMAD)
13.)	Root Mean Squared (RMS)
14.)	Skewness
15.)	Excess Kurtosis
16.)	Variance
17.)	Intensity Histogram Uniformity
	**Local Binary pattern (LBP) Features-m1**
1.)	Energy
2.)	Intensity Histogram Entropy
3.)	Minimum
4.)	10th Percentile
5.)	90th Percentile
6.)	Maximum
7.)	Mean
8.)	Median
9.)	Interquartile Range
10.)	Range
11.)	Mean Absolute Deviation (MAD)
12.)	Robust Mean Absolute Deviation (rMAD)
13.)	Root Mean Squared (RMS)
14.)	Skewness
15.)	Excess Kurtosis
16.)	Variance
17.)	Intensity Histogram Uniformity
	**Local Binary pattern (LBP) Features-m2**
1.)	Energy
2.)	Intensity Histogram Entropy
3.)	Minimum

4.)	10th Percentile
5.)	90th Percentile
6.)	Maximum
7.)	Mean
8.)	Median
9.)	Interquartile Range
10.)	Range
11.)	Mean Absolute Deviation (MAD)
12.)	Robust Mean Absolute Deviation (rMAD)
13.)	Root Mean Squared (RMS)
14.)	Skewness
15.)	Excess Kurtosis
16.)	Variance
17.)	Intensity Histogram Uniformity
	**Local Binary pattern (LBP) Features-kurtosis**
1.)	Energy
2.)	Intensity Histogram Entropy
3.)	Minimum
4.)	10th Percentile
5.)	90th Percentile
6.)	Maximum
7.)	Mean
8.)	Median
9.)	Interquartile Range
10.)	Range
11.)	Mean Absolute Deviation (MAD)
12.)	Robust Mean Absolute Deviation (rMAD)
13.)	Root Mean Squared (RMS)
14.)	Skewness
15.)	Excess Kurtosis

16.)	Variance
17.)	Intensity Histogram Uniformity
	**Gray Level Co-occurrence Matrix (GLCM) Features**
1.)	Autocorrelation
2.)	Joint Average
3.)	Cluster Prominence
4.)	Cluster Shade
5.)	Cluster Tendency
6.)	Contrast
7.)	Correlation
8.)	Difference Average
9.)	Difference Entropy
10.)	Difference Variance
11.)	Joint Energy (IBSI: Angular Second Moment)
12.)	Joint Entropy
13.)	Informal Measure of Correlation (IMC) 1
14.)	Informal Measure of Correlation (IMC) 2
15.)	Inverse Difference Moment (IDM)
16.)	Inverse Difference Moment Normalized (IDMN)
17.)	Inverse Difference (ID)
18.)	Inverse Difference Normalized (IDN)
19.)	Inverse Variance
20.)	Maximum Probability (IBSI: Joint maximum)
21.)	Sum Entropy
22.)	Sum of Squares (IBSI: Sum of Squares)
23.)	Maximal Correlation Coefficient (MCC)
	**Gray Level Size Zone Matrix (GLSZM) Features**
1.)	Small Area Emphasis (SAE)
2.)	Large Area Emphasis (LAE)
3.)	Gray Level Non-Uniformity (GLN)

4.)	Gray Level Non-Uniformity Normalized (GLNN)
5.)	Size-Zone Non-Uniformity (SZN)
6.)	Size-Zone Non-Uniformity Normalized (SZNN)
7.)	Zone Percentage (ZP)
8.)	Gray Level Variance (GLV)
9.)	Zone Variance (ZV)
10.)	Zone Entropy (ZE)
11.)	Low Gray Level Zone Emphasis (LGLZE)
12.)	High Gray Level Zone Emphasis (HGLZE)
13.)	Small Area Low Gray Level Emphasis (SALGLE)
14.)	Small Area High Gray Level Emphasis (SAHGLE)
15.)	Large Area Low Gray Level Emphasis (LALGLE)
16.)	Large Area High Gray Level Emphasis (LAHGLE)
	**Gray Level Run Length Matrix (GLRLM) Features**
1.)	Short Run Emphasis (SRE)
2.)	Long Run Emphasis (LRE)
3.)	Gray Level Non-Uniformity (GLN)
4.)	Gray Level Non-Uniformity Normalized (GLNN)
5.)	Run Length Non-Uniformity (RLN)
6.)	Run Length Non-Uniformity Normalized (RLNN)
7.)	Run Percentage (RP)
8.)	Gray Level Variance (GLV)
9.)	Run Variance (RV)
10.)	Run Entropy (RE)
11.)	Low Gray Level Run Emphasis (LGLRE)
12.)	High Gray Level Run Emphasis (HGLRE)
13.)	Short Run Low Gray Level Emphasis (SRLGLE)
14.)	Short Run High Gray Level Emphasis (SRHGLE)
15.)	Long Run Low Gray Level Emphasis (LRLGLE)
16.)	Long Run High Gray Level Emphasis (LRHGLE)

	**Neighbouring Gray Tone Difference Matrix (NGTDM) Features**
1.)	Coarseness
2.)	Contrast
3.)	Busyness
4.)	Complexity
5.)	Strength
	**Gray Level Dependence Matrix (GLDM) Features**
1.)	Small Dependence Emphasis (SDE)
2.)	Large Dependence Emphasis (LDE)
3.)	Gray Level Non-Uniformity (GLN)
4.)	Dependence Non-Uniformity (DN)
5.)	Dependence Non-Uniformity Normalized (DNN)
6.)	Gray Level Variance (GLV)
7.)	Dependence Variance (DV)
8.)	Dependence Entropy (DE)
9.)	Low Gray Level Emphasis (LGLE)
10.)	High Gray Level Emphasis (HGLE)
11.)	Small Dependence Low Gray Level Emphasis (SDLGLE)
12.)	Small Dependence High Gray Level Emphasis (SDHGLE)
13.)	Large Dependence Low Gray Level Emphasis (LDLGLE)
14.)	Large Dependence High Gray Level Emphasis (LDHGLE)

TABLE S4
Extension of Table 3

AUROC, BA, F1 Score and MCC for the Support Vector Machine (SVM) and Random Forest Classifier (RFC) models trained on both segmentation modes, Gross Tumour Volume (GTV) and Clinical Target Volume (CTV).

Model	Segmentation	AUC	BA	F1	MCC
SVM	GTV	0.58 ± 0.01	0.54 ± 0.02	0.33 ± 0.03	0.08 ± 0.04
	CTV	0.61 ± 0.01	0.57 ± 0.02	0.36 ± 0.03	0.13 ± 0.04
RFC	GTV	0.55 ± 0.01	0.52 ± 0.02	0.29 ± 0.03	0.05 ± 0.04
	CTV	0.62 ± 0.01	0.58 ± 0.02	0.37 ± 0.03	0.15 ± 0.04

* Data is given as mean ± 1.96 standard errors for a 95% confidence interval.

TABLE S5
Extension of Table 4

AUROC, BA, F1 Score and MCC for the SVM, RFC and Logistic Regression (LR) models trained on semantic, clinical, and SINS features.

Model	Data	AUROC	BA	F1	MCC
SVM	CTV	0.61 ± 0.01	0.57 ± 0.02	0.36 ± 0.03	0.13 ± 0.04
RFC		0.62 ± 0.01	0.58 ± 0.02	0.37 ± 0.03	0.15 ± 0.04
SVM	Semantic	0.61 ± 0.01	0.57 ± 0.02	0.38 ± 0.03	0.13 ± 0.04
RFC		0.63 ± 0.01	0.58 ± 0.02	0.39 ± 0.03	0.16 ± 0.04
SVM	Clinical	0.80 ± 0.01	0.72 ± 0.03	0.56 ± 0.05	0.43 ± 0.06
RFC		0.79 ± 0.01	0.73 ± 0.03	0.58 ± 0.05	0.44 ± 0.06
LR	SINS	0.65 ± 0.01	0.58 ± 0.03	0.36 ± 0.05	0.16 ± 0.06
	SINS (binary)	0.54 ± 0.01	0.52 ± 0.03	0.19 ± 0.05	0.04 ± 0.06

* Data is given as mean ± 1.96 standard errors for a 95% confidence interval.

TABLE S6
Extension of Table 5

AUROC, BA, F1 Score and MCC for the SVM and RFC models trained on the different combined datasets.

Model	Data	AUROC	BA	F1	MCC
SVM	CTV + SINS	0.61 ± 0.01	0.57 ± 0.02	0.36 ± 0.04	0.13 ± 0.04
	CTV + Clinical	0.75 ± 0.01	0.69 ± 0.02	0.52 ± 0.03	0.35 ± 0.04
	Semantic + SINS	0.62 ± 0.01	0.58 ± 0.02	0.39 ± 0.03	0.15 ± 0.04
	Semantic + Clinical	0.68 ± 0.01	0.63 ± 0.02	0.45 ± 0.03	0.24 ± 0.04
	CTV + SINS + Clinical	0.74 ± 0.01	0.68 ± 0.02	0.50 ± 0.03	0.33 ± 0.05
	CTV + SINS + Clinical + Semantic	0.67 ± 0.01	0.62 ± 0.02	0.44 ± 0.03	0.22 ± 0.04
RFC	CTV + SINS	0.60 ± 0.01	0.57 ± 0.02	0.36 ± 0.04	0.13 ± 0.04
	CTV + Clinical	0.68 ± 0.01	0.61 ± 0.02	0.42 ± 0.03	0.21 ± 0.04
	Semantic + SINS	0.65 ± 0.01	0.59 ± 0.02	0.40 ± 0.03	0.17 ± 0.04
	Semantic + Clinical	0.72 ± 0.01	0.64 ± 0.02	0.48 ± 0.03	0.27 ± 0.04
	CTV + SINS + Clinical	0.67 ± 0.01	0.61 ± 0.02	0.42 ± 0.03	0.21 ± 0.04

	CTV + SINS + Clinical + Semantic	0.67 ± 0.01	0.61 ± 0.02	0.44 ± 0.03	0.20 ± 0.04

* Data is given as mean ± 1.96 standard errors for a 95% confidence interval.

TABLE S7
AUROC, BA, F1 Score and MCC for the SVM and RFC models trained on clinical and SINS features.

Model	Data	AUROC	BA	F1	MCC
SVM	Clinical + SINS	0.73 ± 0.01	0.68 ± 0.03	0.52 ± 0.04	0.32 ± 0.05
RFC		0.75 ± 0.01	0.68 ± 0.03	0.53 ± 0.04	0.35 ± 0.05

* Data is given as mean ± 1.96 standard errors for a 95% confidence interval.

TABLE S8
Feature importance table for SVM and RFC models trained on CTV features.

Importance in RFC is measured by the mean decrease in impurity. Feature importance in SVM is not possible to estimate given that most kernels were non-linear. Score is calculated by multiplying the importance of a feature by their frequency.

SVM Feature Name	% Chosen		RFC Feature Name	% Chosen	Importance	Score
GLSZM - LAHGLE	94.8		GLCM - DE	94.4	0.071	0.067
GLCM - DE	92		GLCM - Cluster Shade	90.8	0.0699	0.0635
GLCM - Cluster Shade	90		GLSZM - LAHGLE	96.8	0.0648	0.0627
GLRLM - SRE	81.6		Shape - Maximum 2D Diameter Row	78.8	0.0751	0.0592
Shape - Maximum 2D Diameter Row	76.8		GLRLM - SRE	87.2	0.0626	0.0546
GLRLM - LRE	63.6		GLRLM - LRE	64.8	0.0669	0.0434
Shape - Elongation	54.4		GLCM - IDMN	54	0.0675	0.0365
GLSZM - SZNN	54		Shape - Elongation	51.2	0.0704	0.036
GLCM - IDMN	53.6		GLRLM - RLNN	52.8	0.0632	0.0334
GLRLM - RLNN	51.6		GLSZM - SZNN	52.4	0.0633	0.0332
GLSZM - SAE	45.6		Shape - Surface Volume Ratio	39.2	0.0713	0.0279
GLDM - LDLGLE	40.8		GLSZM - SAE	44	0.059	0.026
GLDM - LDHGLE	37.2		GLDM - LDLGLE	42	0.0616	0.0259
GLSZM - GLNN	35.2		GLDM - LDE	35.6	0.0697	0.0248
GLCM - Cluster Prominence	34.4		GLCM - Cluster Prominence	35.6	0.0692	0.0246

TABLE S9
Feature importance table for SVM and RFC models trained on clinical features.

SVM Feature Name	% Chosen		RFC Feature Name	% Chosen	Importance	Score
Age	100		Tumour Type: Others	100	0.2229	0.2229
KPS	100		Tumour Type: Breast Cancer	100	0.2218	0.2218
Opiate Medication	100		Tumour Type: NSCLC	100	0.1969	0.1969

Tumour Type: Breast Cancer	100		Opiate Medication	100	0.1508	0.1508
Tumour Type NSCLC	100		KPS	100	0.1235	0.1235
Tumour Type: Others	100		Age	100	0.0841	0.0841

TABLE S10
Feature importance table for SVM and RFC models trained on semantic features.

SVM Feature Name	% Chosen		RFC Feature Name	% Chosen	Importance	Score
Vertebral body collapse: <50% collapse	100		GTV - Classification: Body + bilateral pedicle/transverse processBody	98.4	0.0775	0.0763
Posterolateral involvement of the spinal elements: None of the above	100		Imaging - Bone reaction: Lytic	98	0.0743	0.0728
Location: Mobile	99.2		Posterolateral involvement of the spinal elements: Bilateral	94.4	0.0753	0.0711
Vertebral body collapse: >50% collapse	98.8		GTV - Classification: Body + bilateral pedicle/transverse	93.2	0.0729	0.0679
Imaging - Bone reaction: Lytic	97.6		Soft tissue component: Yes	84.4	0.0778	0.0656
GTV - Classification: Body + unilateral pedicle	97.6		Vertebral body collapse: >50% collapse	99.2	0.0653	0.0647
Posterolateral involvement of the spinal elements: Bilateral	96		Posterolateral involvement of the spinal elements: None of the above	100	0.0644	0.0644
GTV - Classification: Body + bilateral pedicle/transverse processBody	94.4		Soft tissue component: No	82.8	0.0774	0.0641
Location: Semirigid	88.4		Imaging - Bone reaction: Mixed	49.2	0.1105	0.0544
Soft tissue component: Yes	86		Vertebral body collapse: <50% collapse	100	0.0536	0.0536
Soft tissue component: No	84.8		Posterolateral involvement of the spinal elements: Unilateral	71.2	0.0708	0.0504
Posterolateral involvement of the spinal elements: Unilateral	64		Imaging - Bone reaction: Blastic	39.2	0.1115	0.0437
Vertebral body collapse: No collapse with >50% body involved	52.8		Location: Mobile	98	0.0426	0.0418
GTV - Classification: Unilateral pedicle	51.6		GTV - Classification: Unilateral pedicle	47.6	0.0747	0.0356

Vertebral body collapse: None of the above	48		Location: Semirigid	88.4	0.0397	0.0351

TABLE S11
Feature importance table for SVM and RFC models trained on CTV, clinical, SINS and semantic features.

SVM Feature Name	% Chosen		RFC Feature Name	% Chosen	Importance	Score
Tumour Type: Breast Cancer	100		Posterolateral involvement of the spinal elements: None of the above	94	0.0714	0.0671
Tumour Type: Others	93.6		Tumour Type: Breast Cancer	99.2	0.0598	0.0594
Posterolateral involvement of the spinal elements: None of the above	90.8		GLSZM - LAHGLE	89.6	0.0653	0.0585
GLSZM - LAHGLE	88.4		Tumour Type: Others	93.2	0.061	0.0569
GTV - Classification: Unilateral pedicle	84		Vertebral body collapse: <50% collapse	79.2	0.0716	0.0567
Vertebral body collapse: <50% collapse	78.4		GLCM - Cluster Shade	78	0.0721	0.0562
GLCM - Cluster Shade	76		GTV - Classification: Unilateral pedicle	83.2	0.0627	0.0521
Location: Mobile	70.4		Location: Mobile	70	0.0721	0.0505
GLDM - LDHGLE	68		GLDM - LDHGLE	72.4	0.067	0.0485
GLCM - DE	58		Imaging - Bone reaction: Lytic	61.6	0.0699	0.0431
Imaging - Bone reaction: Lytic	57.6		Location: Semirigid	50	0.0663	0.0331
Location: Semirigid	46.8		GLCM - DE	48.8	0.0668	0.0326
GTV - Classification: Body + bilateral pedicle/transverse processBody	34		GTV - Classification: Body + bilateral pedicle/transverse processBody	36.4	0.0677	0.0246
Posterolateral involvement of the spinal elements: Bilateral	31.6		Posterolateral involvement of the spinal elements: Bilateral	34.4	0.0652	0.0224
Vertebral body collapse: >50% collapse	30.4		Vertebral body collapse: >50% collapse	27.2	0.0727	0.0198

TABLE S12
Averaged optimal hyperparameter values for the three best models.

The values shown have been averaged across the 250 final models. In the cases where an average is not possible (e.g., categorical parameters), the mode has been used. In the cases where a parameter does not make sense or alters the model in any way (e.g., *gamma* or *degree* for a linear kernel in SVM), it has been accordingly excluded.

Model	max_features	max_depth	min_samples_split	min_samples_leaf	Bootstrap	Criterion

CTV radiomics model (RFC)	'auto'	503,22	5,21	2,92	TRUE	'gini'
Clinical model (SVM)	**C**	**Kernel**	**Degree**	**Gamma**		
	0,5	poly'	4,54	8,62		
Combined CTV and clinical model (SVM)	**C**	**Kernel**	**Degree**	**Gamma**		
	0,43	poly'	3,57	4,73		

SUPPLEMENTAL FIGURES

FIGURE S1
Patients workflow

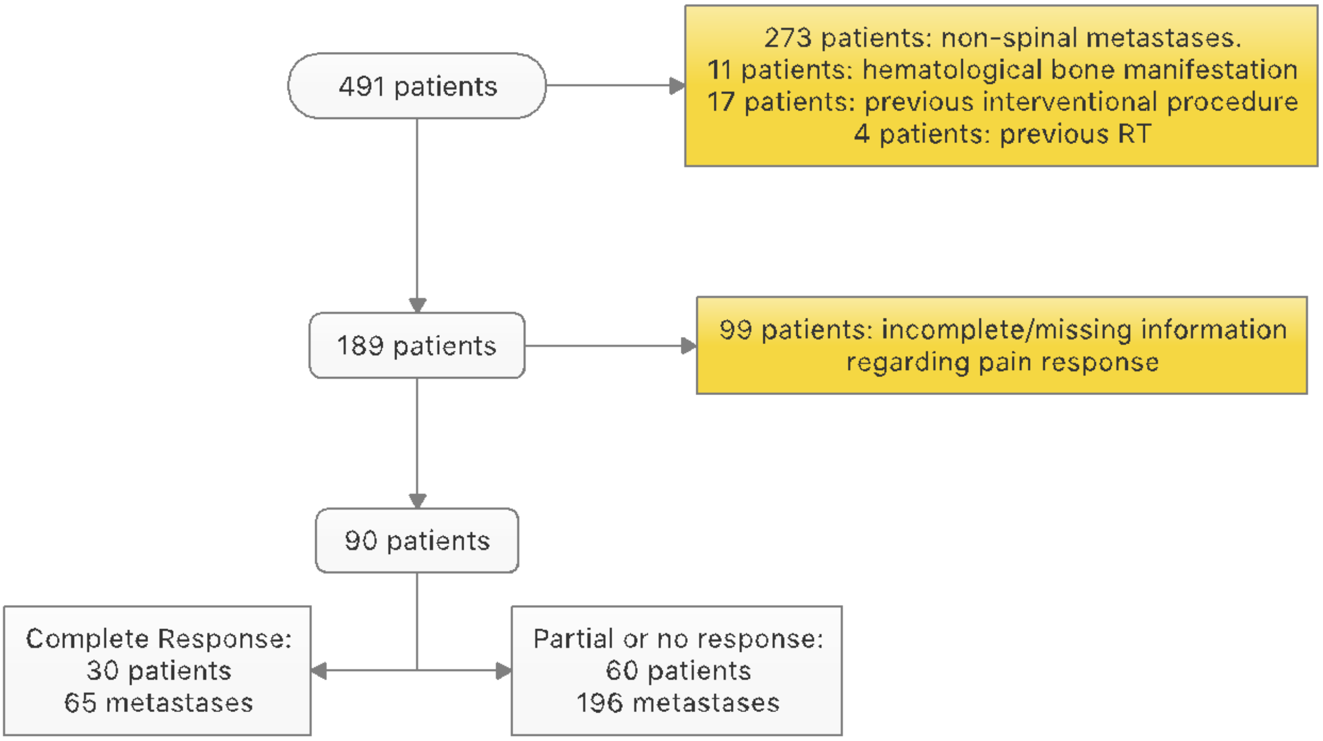

FIGURE S2
ROC and Calibration Curves: Extension of Figure 2

Receiver operator characteristic (ROC) and Calibration curves for the comparisons of the remaining models, from Table 3 and Table 5, that are not shown in Figure 2.

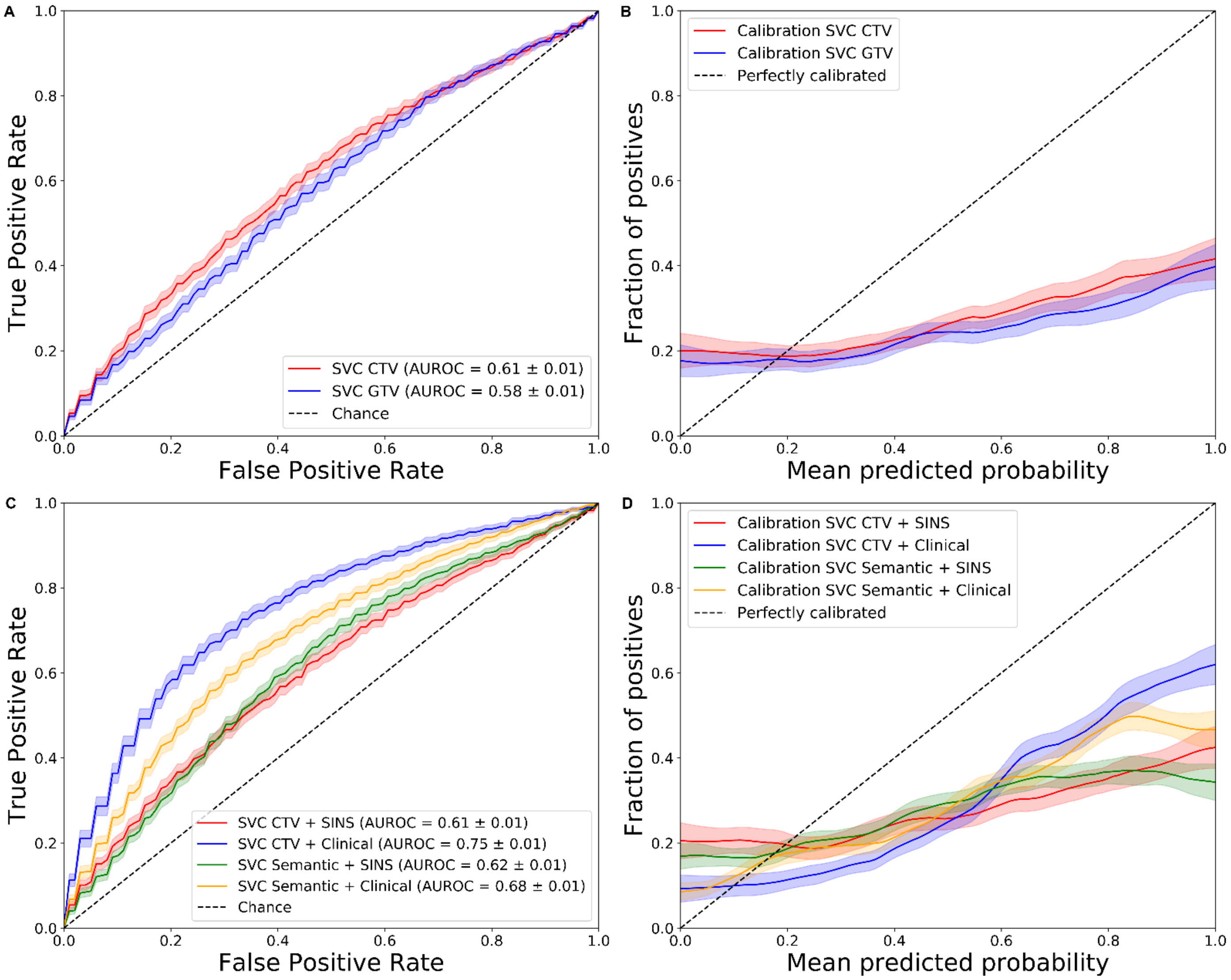
A
B
C
D
True Positive Rate
False Positive Rate
Fraction of positives
Mean predicted probability
SVC CTV (AUROC = 0.61 ± 0.01)
SVC GTV (AUROC = 0.58 ± 0.01)
Chance
Calibration SVC CTV
Calibration SVC GTV
Perfectly calibrated
SVC CTV + SINS (AUROC = 0.61 ± 0.01)
SVC CTV + Clinical (AUROC = 0.75 ± 0.01)
SVC Semantic + SINS (AUROC = 0.62 ± 0.01)
SVC Semantic + Clinical (AUROC = 0.68 ± 0.01)
Chance
Calibration SVC CTV + SINS
Calibration SVC CTV + Clinical
Calibration SVC Semantic + SINS
Calibration SVC Semantic + Clinical
Perfectly calibrated

FIGURE S3
Averaged Confusion Matrix for the best performing radiomics model

Confusion Matrix that averages the classification performances of the 250 best radiomics models (RFC trained on CTV radiomics features).

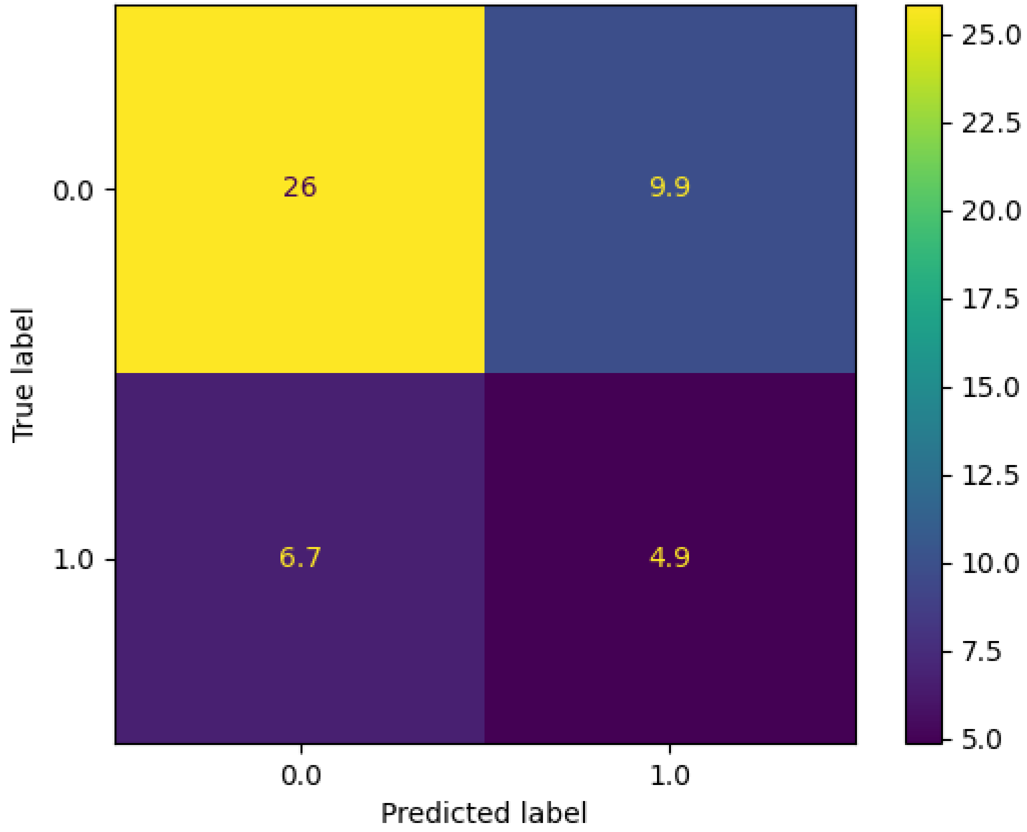

FIGURE S4
Averaged Confusion Matrix for the best performing model overall

Confusion Matrix that averages the classification performances of the 250 best models overall (RFC trained on CTV radiomics features).

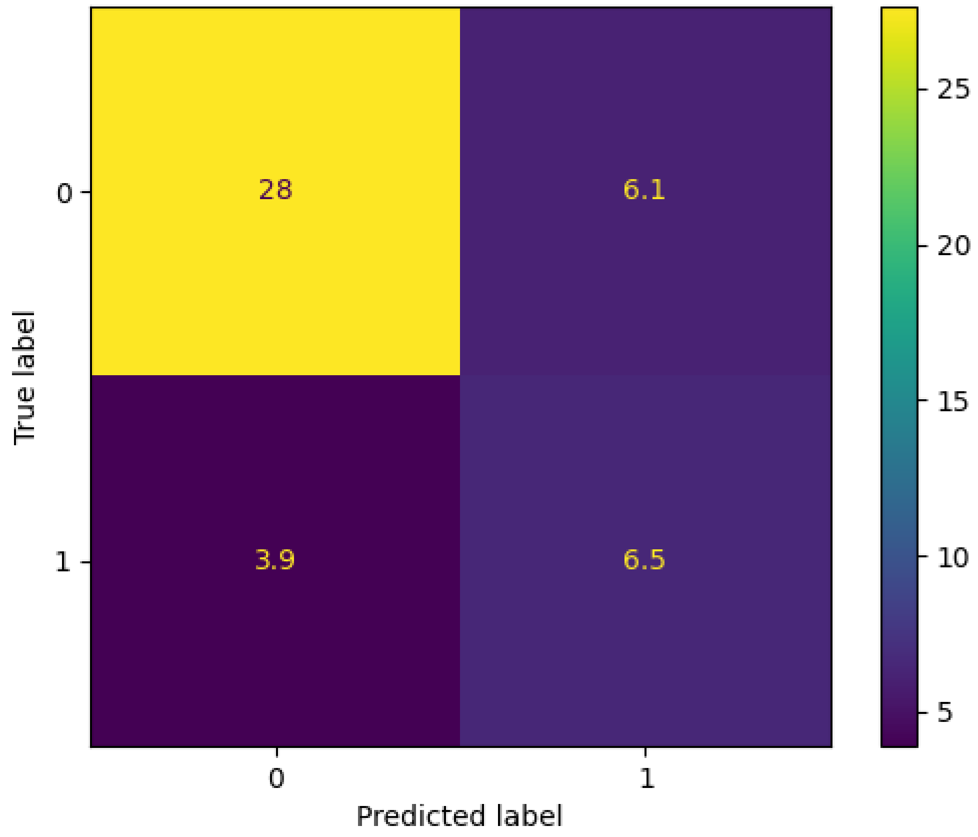

References

1. Zwanenburg, A.; Vallières, M.; Abdalah, M.A.; Aerts, H.J.W.L.; Andrearczyk, V.; Apte, A.; Ashrafinia, S.; Bakas, S.; Beukinga, R.J.; Boellaard, R.; et al. The Image Biomarker Standardization Initiative: Standardized Quantitative Radiomics for High-Throughput Image-based Phenotyping. *Radiology* **2020**, 191145, doi:10.1148/radiol.2020191145.
2. van Griethuysen, J.J.M.; Fedorov, A.; Parmar, C.; Hosny, A.; Aucoin, N.; Narayan, V.; Beets-Tan, R.G.H.; Fillion-Robin, J.-C.; Pieper, S.; Aerts, H.J.W.L. Computational Radiomics System to Decode the Radiographic Phenotype. *Cancer Res.* **2017**, *77*, e104-e107, doi:10.1158/0008-5472.CAN-17-0339.
